# All-cause mortality, adverse events and associated factors following endoscopic retrograde cholangiopancreatography for benign indications in England

**DOI:** 10.1055/a-2865-3052

**Published:** 2026-06-08

**Authors:** Umair Kamran, Philip R. Harvey, Ben Coupland, Jemma Mytton, Kofi W. Oppong, Nigel Trudgill

**Affiliations:** 1 HPB Unit and Department of Gastroenterology 5983 Newcastle Upon Tyne Hospitals NHS Foundation TrustNewcastle Upon TyneUnited Kingdom of Great Britain and Northern Ireland; 2 Department of Gastroenterology 8692 Royal Wolverhampton Hospitals NHS TrustWolverhamptonUnited Kingdom of Great Britain and Northern Ireland; 3 Department of Health Informatics 1732 University Hospitals Birmingham NHS Foundation TrustBirminghamEnglandUnited Kingdom of Great Britain and Northern Ireland; 4 Institute of Translational and Clinical Research 5994 Newcastle UniversityNewcastle upon TyneEnglandUnited Kingdom of Great Britain and Northern Ireland; 5 Department of GastroenterologySandwell and West Birmingham NHS TrustWest BromwichUnited Kingdom of Great Britain and Northern Ireland; 6 Department of Cancer and Genomic Sciences 1724 University of BirminghamBirminghamEnglandUnited Kingdom of Great Britain and Northern Ireland

**Keywords:** Pancreatobiliary (ERCP/PTBD), Stones, Strictures, ERC topics

## Abstract

**Background and study aims:**

Population-based data on outcomes of endoscopic retrograde cholangiopancreatography (ERCP) for benign indications are limited. We examined the impact of changing patient demographics on ERCP outcomes.

**Patients and methods:**

Adults undergoing first ERCP for benign indications between 2003 and 2020 in England were identified in Hospital Episode Statistics. All-cause mortality within 30 days and adverse event rates within 7 days were calculated. Multivariable logistic regression analysis explored factors associated with all-cause mortality.

**Results:**

A total of 283,972 patients were included; 63.4% female; median age 67 years (interquartile range 50–79). Twenty-five percent of ERCPs in 2019/2020 were performed in patients with a Charlson comorbidity score ≥ 5, compared with 8% in 2003/2004. Three percent of patients who underwent day case ERCP had an emergency readmission with pancreatitis. Thirty-day all-cause mortality fell by 31% over the study period. Mortality was associated with age (age quintile ≥ 82 years; odds ratio 19.22; 95% confidence interval [CI] 15.59–23.69 compared with 18–46 years), male sex (1.21; CI 1.14–1.27), Black or Black British ethnicity (1.42; CI 1.07–1.90), increasing comorbidity score ≥ 5 (3.35; CI 3.14–3.57), and increasing provider annual ERCP volume (volume ≥ 151; 1.08; CI 1.01–1.16). Elective ERCP (0.30; CI 0.28–0.33) and those in recent years were associated with lower mortality (2019/2020 compared with 2003/2004 (0.40; CI 0.34–0.48). Statistically significant variations in 30-day all-cause mortality were observed between ERCP providers.

**Conclusions:**

Despite the increasing age and comorbidities of patients undergoing ERCP for benign indications, 30-day all-cause mortality fell by 31% in recent years. All-cause mortality was associated with age, males, comorbidities, black ethnicity, emergency ERCP, and increasing provider volume.

## Introduction

Endoscopic retrograde cholangiopancreatography (ERCP) is recognized to be associated with a number of adverse events (AEs) including pancreatitis, cholangitis, bleeding, and perforation 1 2 3 . The procedure and its potential AEs are associated with a significant risk of death 4 5 6 . ERCP is undertaken for both benign and malignant indications. Technical success rates, AEs, and need for repeat procedures differ for benign and malignant indications. Previous studies have demonstrated that post ERCP 30-day mortality is much higher following ERCP to palliate malignant biliary obstruction at 19% 7 and lower among patients undergoing ERCP for benign indications 8 . A Swedish registry analysis of data from 2005 to 2008 identified a 30-day mortality rate of 2.2% following ERCP for benign indications 9 .

Current evidence for differences in outcomes following ERCP by volume of procedures is conflicting. Studies have suggested that mortality is lower and procedure success rates higher at centers performing a larger number of ERCPs 9 10 11 . However, this is not consistent across all studies, with some larger analyses not demonstrating any difference in outcomes with procedure volume 8 . Case mix may contribute to this variation in findings. Mortality following ERCP is higher when the procedure is undertaken for unresectable cancer, and a volume effect has been observed in this setting 7 .

The UK population is aging with recent Office for National Statistics (ONS) data showing that 18% are older than 65 and 2.4% older than 85 years of age 12 . By comparison, in 1996, 15.8% were older than age 65 years and in 2036 this age group is expected to be 23.9% of the population 12 . Therefore, current and future ERCP practice in England will include patients who are older and who have more comorbidity than previously, both of which relate to AEs and mortality following ERCP 8 .

The aims of this study were to examine 30-day all-cause mortality rates following an ERCP for benign indications in England over time, variation in all-cause mortality rates among ERCP providers, and factors associated with all-cause mortality. We have also examined rates of AE events within 7 days of ERCP.

## Patients and methods

### Hospital episode statistics

Hospital episode statistics (HES) is an administrative database including all episodes of National Health Service (NHS) hospital treatment within England, including outpatient procedures. Data are derived from administrative processes within the NHS and available for secondary research 13 . Patients are assigned a unique identification number, allowing their hospital episodes to be linked. Diagnoses are described using International Classification of Diseases 10 (ICD-10) codes, and procedures by Office of Population Census and Surveys Classification of Interventions and Procedures version 4 (OPCS4) codes. Demographic and administrative data are also available. Records are linked to the Office of National Statistics (ONS) to provide mortality data and details of death certification. HES is governed by a data-sharing agreement prohibiting publication of any information that may be identifiable, with any data item of five or less suppressed from publication.

### Subject cohort

Patients were identified by their first OPCS4 code for ERCP (Supplementary Table 1). Any subject with an ICD-10 code for cancer within 2 years either before or after ERCP or cited on their death certificate were excluded. Further exclusion criteria included: any subject with a liver transplant or autoimmune biliary disease, patients aged less than 18 years or resident outside of England, and patients with incomplete demographic data.

### Data validation

The total number of ERCPs meeting the study criteria was calculated at Sandwell and West Birmingham NHS trust (SWBNHST) between April 1, 2014 and March 31, 2015 using local endoscopy reporting software. This was compared with the equivalent figures over the same time period within HES.

### Data extraction

Data items extracted included demographic details such as age, sex, ethnicity, deprivation quintile (index of multiple deprivations 2010 (IMD)) and Charlson comorbidity score. Age was included as a categorical variable and grouped into quintiles (18–46, 47–62, 63–72, 73–81 and ≥ 82 years). Ethnicity was classified into White, Asian or Asian British, Black or Black British, mixed ethnicity and other minority ethnicities. The comorbidity score was generated from ICD-10 codes identified within HES data, a strategy that has been previously validated 14 15 . Deprivation level was calculated using an aggregate score for English Lower Layer Super Output Areas (LSOA), based on employment status, income, crime levels, and living environment 16 . Deprivation was categorized into quintiles, with 1 the most deprived and 5 the least deprived. The year of ERCP was collected, as was admission type (defined as emergency, elective, or other) for the procedure. Provider annual volume of benign ERCPs was estimated from HES records and providers grouped into three categories based on their annual volume; 1 to 100, 101 to 150, and > 150.

### Adverse events and mortality

All-cause mortality within 30 days of ERCP was identified using linked ONS data. Data were extracted on AEs within 7 days of ERCP including gastrointestinal bleeding, perforation, acute pancreatitis, cholangitis, and sedation related (Supplementary Table 1). Because it can be difficult to establish from coding records if pancreatitis or cholangitis were the indication for or an AE of ERCP if these were coded within the same inpatient episode, rates of pancreatitis and cholangitis following ERCP were only examined for patients who underwent ERCP as day cases and were subsequently admitted as an emergency with acute pancreatitis or cholangitis within 7 days of their ERCP for the last 8 years of study period. For all other AEs, rates were reported for the whole cohort and included all incidences within 7 days of ERCP. Data were also extracted for repeat ERCP procedures within 90 days (either in the same hospital admission or at a later stage) and emergency readmission within 30 days.

### Statistical analysis

Categorical variables were summarized as number and percentages. Abbreviated demographics including sex, age and comorbidity score are reported by the year of index ERCP for years 3/4, 9/10, 14/15, and 19/20 to identify how the population of patients undergoing ERCP has changed in England.

A logistic regression model explored patient and endoscopy provider characteristics associated with 30-day all-cause mortality. All exploratory variables were included as categorical variables and included sex, age, ethnicity, deprivation, procedure type (elective or emergency procedure), year of ERCP, Charlson comorbidity score, and provider annual ERCP volume. Patients requiring early repeat ERCP may represent a cohort with more complex disease and a sensitivity analysis was performed to examine associations with 30-day all-cause mortality after excluding patients who underwent repeat ERCP within 90 days.

Funnel plots were produced to assess variation in unadjusted and adjusted 30-day all-cause mortality rates in relation to benign ERCP volume for each provider. These are constructed as scatter plots with superimposed control limits, which represent two and three standard deviations from the mean. Providers falling outside these control limits have 30-day all-cause mortality rates that are statistically different from the national mean, following adjusting for sex, age, ethnicity, deprivation, comorbidity score, procedure type, and year of ERCP. Because mortality has fallen in recent years, a breakdown of 30-day mortality rates based on the last 3 years of the study period and stratified by patient age and sex was extracted to inform patient procedural consent in the UK.

Data were analyzed using Stataversion 15 (Stata Corp. 2017. Stata Statistical Software: Release 15. College Station, Texas, United States: StataCorp LP) 17 , two-sided *P* < 0.05 were considered statistically significant.

### Ethics

Ethics approval was not required because a data-sharing agreement with NHS Digital is in place for the purpose of service evaluation. The study was registered with the clinical audit and research department at SWBNHST and the health informatics department at University Hospitals Birmingham NHS Trust.

## Results

### Validation

Validation identified 123 patients at SWBNHST who met the inclusion criteria for this study. HES identified 119 in the same time period (96.7% concordance).

### Patient cohort

**Fig. 1:**
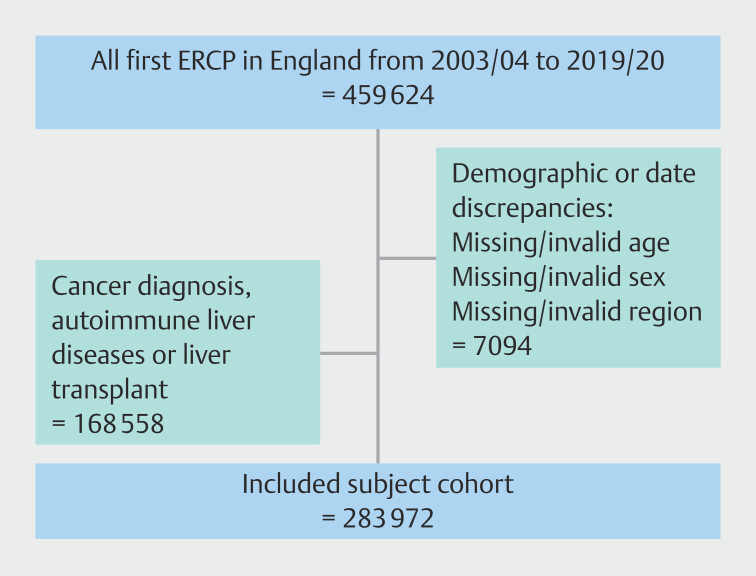
Study flowchart of exclusions and inclusions.

There were 459,624 first ERCPs performed between April 2003 and April 2020, of whom 168,558 patients (36.7%) were excluded due to either cancer or a concurrent liver transplant or autoimmune diagnosis. A further 7,094 (1.5%) were excluded due to data quality concerns. Therefore, 283,972 patients were included in the study ( Fig. 1 ).

### Demographics

Demographic details are presented in Table 1 . Of the patients, 180,696 (63.6%) were female and 257,459 (90.7%) were White. Charlson comorbidity score was 0 in 190,888 patients (67.2%), 1 to 4 in 42,720 patients (15.0%), and ≥ 5 in 50,364 (17.7%). Median age was 67 years (interquartile range [IQR] 50–79). Of included ERCPs, 153,791 (54.2%) were identified as occurring during an emergency admission.

**Table:** **Table 1** Demographic details of study patients and associated all-cause mortality.

Factors		Number of patients	Percentage	30-day mortality	Percentage
Sex	Male	103276	36.4%	2398	2.3%
	Female	180696	63.6%	3036	1.7%
Age quintiles	18–46	57036	20.1%	94	0.2%
	47–62	60417	21.3%	334	0.6%
	63–72	53678	18.9%	671	1.3%
	73–81	58124	20.5%	1461	2.5%
	≥ 82	54717	19.3%	2874	5.3%
Deprivation	1 (most deprived)	62443	22.0%	1219	2.0%
	2	58435	20.6%	1106	1.9%
	3	57661	20.3%	1099	1.9%
	4	54651	19.3%	1086	2.0%
	5 (least deprived)	49043	17.3%	904	1.8%
	Unknown	1739	0.6%	20	1.2%
Ethnicity	White	257459	90.7%	4823	1.9%
	Black or Black British	3666	1.3%	50	1.4%
	Asian or Asian British	11096	3.9%	176	1.6%
	Mixed	1204	0.4%	12	1.0%
	Other	1652	0.6%	12	0.7%
	Unknown	8895	3.1%	361	4.1%
Procedure type	Emergency	153791	54.2%	4551	3.0%
	Elective	127118	44.8%	780	0.6%
	Unknown/other	3063	1.1%	103	3.4%
Charlson comorbidity score	0	190888	67.2%	1822	1.0%
	1–4	42720	15.0%	620	1.5%
	≥ 5	50364	17.7%	2992	5.9%
Provider annual ERCP volume	1–100	75332	26.5%	1377	1.8%
	101–150	97505	34.3%	1805	1.9%
	≥ 151	111135	39.1%	2252	2.0%
Year of ERCP	2003/2004	14093	4.9%	297	2.1%
	2004/2005	13323	4.7%	303	2.3%
	2005/2006	14197	5.0%	320	2.3%
	2006/2007	14343	5.1%	335	2.3%
	2007/2008	14409	5.1%	334	2.3%
	2008/2009	15210	5.4%	334	2.2%
	2009/2010	15517	5.5%	291	1.9%
	2010/2011	16269	5.7%	293	1.8%
	2011/2012	16836	5.9%	323	1.9%
	2012/2013	17100	6.0%	330	1.9%
	2013/2014	18319	6.5%	323	1.8%
	2014/2015	18869	6.6%	368	2.0%
	2015/2016	18701	6.6%	327	1.8%
	2016/2017	18854	6.6%	356	1.9%
	2017/2018	18776	6.6%	315	1.7%
	2018/2019	19476	6.9%	300	1.5%
	2019/2020	19680	6.9%	285	1.5%
Total patients		283972		5434	1.9%
ERCP, endoscopic retrograde cholangiopancreatography.					

Over the study period, the population of patients undergoing ERCP became older and more comorbid. In the first year of the study period (2003/04) 2,321 (16.5%) patients were older than age 81 years, compared with 4,166 (21.2%) in the final year (2019/20) ( *P* < 0.001). Similarly, an increasing proportion of patients with Charlson comorbidity score ≥ 5 was observed, comprising 1,206 patients (8.6%) in the first year compared with 5003 patients (25.4%) in the final year ( *P* < 0.001). The changing demographic data are shown in Table 2 .

**Table:** **Table 2** Change in demographic details of patients undergoing ERCP for benign indications over the study period.

Financial year		2003/2004		2009/2010		2014/2015		2019/2020		Full study cohort	
Demographic		**n**	**%**	**n**	**%**	**n**	**%**	**n**	**%**	**n**	**%**
Sex	Male	4717	33.5%	5534	35.7%	6881	36.5%	7980	40.5%	103276	36.4%
	Female	9376	66.5%	9983	64.3%	11988	63.5%	11700	59.5%	180696	63.6%
Age quintile	18–46	3217	22.8%	3296	21.2%	3850	20.4%	3244	16.5%	57036	20.1%
	47–62	3354	23.8%	3316	21.4%	3910	20.7%	3889	19.8%	60417	21.3%
	63–72	2474	17.6%	2761	17.8%	3621	19.2%	4007	20.4%	53678	18.9%
	73–81	2727	19.4%	3149	20.3%	3785	20.1%	4374	22.2%	58124	20.5%
	>81	2321	16.5%	2995	19.3%	3703	19.6%	4166	21.2%	54717	19.3%
Comorbidity score	0	11769	83.5%	10968	70.7%	11770	62.4%	10946	55.6%	190888	67.2%
	1 to 4	1118	7.9%	2100	13.5%	3207	17.0%	3731	19.0%	42720	15.0%
	≥ 5	1206	8.6%	2449	15.8%	3892	20.6%	5003	25.4%	50364	17.7%
Total		14093		15517		18869		19680		283972	
ERCP, endoscopic retrograde cholangiopancreatography.											

### Adverse events

Three percent (95% confidence interval [CI] 2.8%-3.1%) of the patients who underwent ERCP for benign indications as a day case between 2012/13 and 2019/20 had an emergency readmission with acute pancreatitis within 7 days of their ERCP. Gastrointestinal bleeding was reported in 1.0% (95% CI 0.9%-1.0%) and perforation in 0.4% (95% CI 0.3%-0.4%) of patients post ERCP. The 30-day readmission rate following ERCP in this cohort was 12.5% ( Table 3 ). Following an initial ERCP, 47,140 patients (16.6%) required a further procedure within 90 days. A further ERCP was performed during the same admission in 9,861 patients (3.5%).

**Table:** **Table 3** Adverse events, readmissions and repeat ERCP procedures following ERCP for benign indications.

Adverse event type	Adverse event ^†^	Percentage
Pancreatitis*	2000	3.0%
Cholangitis*	510	0.8%
Gastrointestinal bleeding	2,892	1.0%
Perforation	1,120	0.4%
Sedation-related adverse events	2,661	0.9%
30-day emergency readmission	35,618	12.5%
Repeat ERCP within 90 Days	47,140	16.6%
Repeat ERCP same admission	9,861	3.5%
*The proportion of patients who underwent ERCP as a day case (n = 67,695) between 2012/13 and 2019/20 and had an emergency readmission with ERCP-related adverse events within 7 days. ^†^ Adverse events coded within 7 days unless otherwise specified. ERCP, endoscopic retrograde cholangiopancreatography.		

### All-cause mortality

The overall all-cause mortality rate at 30 days following ERCP was 1.9% (n = 5,434). Mortality varied by demographic characteristics and was 5.3% in patients older than age 81 years and 5.9% in those with a comorbidity score greater than 4. Mortality fell by 31% (95% CI 30%-34%) over the study period (2.1%; 95% CI 1.9%-2.4%) in 2003/2004 to 1.5% (95% CI 1.3%-1.6%) in 2019/2020). This reduction in the all-cause mortality rate was more pronounced in the patients older than age 80 years (Supplementary Fig. 1). A breakdown of 30-day all-cause mortality rates for the last 3 years of the study period, stratified by age and sex, is presented in Supplementary Table 2.

### Logistic regression analysis of associations with 30-day all-cause mortality

On logistic regression analysis, 30-day all-cause mortality was associated with; males (odds ratio [OR] 1.21; 95% CI 1.14–1.27); increasing age – quintile 47–62 (3.19; 2.53–4.01), age 63–72 (6.46; 5.19–8.04), age 73–81 (10.91; 8.83–13.49), and age ≥ 82 (19.22; 15.59–23.69); Charlson comorbidity score 1–4 (1.25; 1.14–1.37) and score ≥ 5 (3.35; 3.14–3.57); Black ethnicity (1.42; 1.07–1.90); provider annual ERCP volume (ERCP volume ≥ 151, 1.08; 1.01–1.16); elective ERCP (0.30; 0.28–0.33); and more recent ERCP relative to the first year included in the study (2019/2020 (0.40; 0.34–0.48). The full regression model is shown in Table 4 .

**Table:** **Table 4** Multivariable logistic regression analysis of factors associated with 30-day all-cause mortality following ERCP for benign indications.

Factors		Odds ratio	95% CI	
Sex	Female	Reference category		
	Male	1.21	1.14	1.27
Age quintile	18–46	Reference category		
	47–62	3.19	2.53	4.01
	63–72	6.46	5.19	8.04
	73–81	10.91	8.83	13.49
	≥ 82	19.22	15.59	23.69
Deprivation	1 (most deprived)	Reference category		
	2	0.95	0.87	1.03
	3	0.93	0.86	1.02
	4	0.99	0.90	1.07
	5 (least deprived)	0.94	0.86	1.03
	Unknown	0.75	0.48	1.18
Ethnicity	White	Reference category		
	Black or Black British	1.42	1.07	1.90
	Asian or Asian British	1.06	0.91	1.24
	Mixed	1.24	0.69	2.22
	Other/unknown	2.62	2.34	2.94
Procedure type	Emergency	Reference category		
	Elective	0.30	0.28	0.33
	Other	1.42	1.16	1.75
Year of ERCP	2003/2004	Reference category		
	2004/2005	1.03	0.87	1.21
	2005/2006	1.03	0.88	1.22
	2006/2007	1.00	0.85	1.18
	2007/2008	0.97	0.83	1.14
	2008/2009	0.90	0.77	1.07
	2009/2010	0.72	0.61	0.85
	2010/2011	0.66	0.56	0.78
	2011/2012	0.67	0.57	0.79
	2012/2013	0.66	0.56	0.78
	2013/2014	0.59	0.50	0.69
	2014/2015	0.65	0.55	0.76
	2015/2016	0.58	0.49	0.68
	2016/2017	0.61	0.52	0.71
	2017/2018	0.52	0.44	0.62
	2018/2019	0.45	0.38	0.54
	2019/2020	0.40	0.34	0.48
Charlson comorbidity score	0	Reference category		
	1–4	1.25	1.14	1.37
	≥ 5	3.35	3.14	3.57
Provider annual ERCP volume	1–100	Reference category		
	101–150	0.95	0.88	1.02
	≥ 151	1.08	1.01	1.16
CI, confidence interval; ERCP, endoscopic retrograde cholangiopancreatography.				

On sensitivity analysis, after excluding patients who underwent repeat ERCP within 90 days, all associations were sustained except provider annual ERCP volume (ERCP volume ≥ 151, 1.06; 0.98–1.14) (Supplementary Table 3).

### Provider variation in 30-day all-cause mortality

**Fig. 2:**
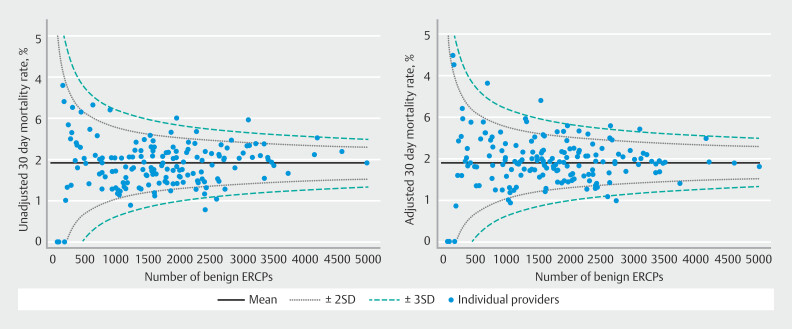
Funnel plots representing variation in unadjusted and adjusted 30-day all-cause mortality rates following ERCP for benign indications in England by provider volume. ERCP, endoscopic retrograde cholangiopancreatography; SD, standard deviation.

Statistically significant variations in unadjusted and adjusted 30-day all-cause mortality rates were observed among ERCP providers. In the unadjusted funnel plot, two ERCP providers were outside the three SD control limits, whereas four providers were outside three 3SD control limits on the adjusted funnel plot ( Fig. 2 ).

## Discussion

In the present study, patients undergoing their first ERCP for benign disease were found to be increasingly older and comorbid. Despite this, encouragingly, all-cause 30-day mortality fell during the study period, suggesting that even with potentially higher-risk patients undergoing ERCP, the impact of improvements in ERCP and peri-procedural care quality have outweighed these demographic changes.

A recent UK report has described a 30-day all-cause mortality rate of 4.2% following ERCP, ranging from 0% to 8.5% among ERCP providers 18 . However, this report did not differentiate between ERCP for benign and malignant indications, and mortality rates were not adjusted for patient demographics and provider volume. All-cause mortality rates following ERCP for malignant biliary obstruction have previously been reported to be as high as 19% 7 , which is in stark contrast to much lower mortality rates of 1.9% following ERCP for benign indications in the present study. These findings indicate that differences in case mix significantly contribute towards variations in 30-day all-cause mortality rates following ERCP and should be taken into account when comparing mortality rates among providers. Although a large relative reduction in mortality of 31% was observed over the study period, the absolute reduction was modest at 0.6%.

Comparing mortality observed in this study to other studies is difficult given the paucity of data for benign disease specifically. In a similar study of Swedish data from 2005 to 2008, 30-day mortality observed was 2.2% 9 . A further Swedish registry analysis, albeit with less certainty regarding ERCP indication, reported similar findings 19 . These studies are in keeping with the present study, in which we observed a mortality rate of 2.3% from 2005 to 2008. The more recent data presented demonstrate that encouragingly mortality has fallen by approximately one-third since that analysis, to 1.5% in 2019/2020, despite patients having increased comorbidity and more advanced age. Reduction in mortality was more pronounced in the most elderly population, with 30-day all-cause mortality reducing to almost half in individuals aged 80 to 90 years and by approximately one-third in those aged > 90 years. This may reflect improved patient selection, advances in peri-procedural care, and refinements in procedural techniques over time. Over the study period, the role of ERCP has undergone significant transformation, moving from often being a diagnostic procedure to being an essentially therapeutic procedure. This change has been largely driven by development and widespread adoption of less invasive diagnostic modalities, such as magnetic resonance cholangiopancreatography and endoscopic ultrasound. These modalities have substantially reduced the need for diagnostic ERCP. Nevertheless, ERCP remains a valuable tool in clinical practice, particularly with expanding knowledge and growing indications for direct cholangioscopy and pancreatoscopy, which continue to enhance its diagnostic and therapeutic potential.

We found an association between ethnicity and post ERCP all-cause mortality, with Black patients having higher all-cause mortality within 30 days of ERCP compared with White patients, despite adjusting for other relevant variables including comorbidity. Several studies have shown that Black ethnicity is an independent predictor of early postoperative mortality across a wide range of surgical procedures; however, to our knowledge, the present study is the first to show this association with early mortality following ERCP 20 21 22 . It was not possible to ascertain the cause of this association from the current study and future studies with access to clinical records should explore variations in ERCP outcomes by ethnicity in more detail.

The present study is the largest analysis of ERCP outcomes and AEs for benign disease in England. Current UK national AE data for ERCP are derived from a national audit from 2007. However, inclusion of patients in this audit was voluntary, subject participation required consent, and if patients presented with AEs to a different hospital, this was not captured 23 . These audit data, therefore, may underestimate AEs. Rates of AEs post ERCP reported in the audit were similar to the present study 23 , and in keeping with other reports 5 24 . Within the described limits of the national audit data, the present study findings suggest that ERCP AEs are adequately coded in the HES database studied.

The rate of pancreatitis was found to be lower than previously published systematic reviews, which reported overall pancreatitis rates between 3.5% and 9.4% 25 26 . The current study reported pancreatitis rates following ERCP for benign indications performed as outpatients only, which may have underestimated overall incidence rates. However, previous reviews may not be entirely reflective of more recent practice. For example, Andriulli et al. included studies published up to 2006 26 . Effective prophylactic measures including rectal administration of nonsteroidal anti-inflammatory drugs (NSAIDs), aggressive hydration, and prophylactic pancreatic stenting have since become established and widely used in the UK 27 . In a more recent review of 108 randomized controlled trials, only patients in the placebo arms were included and the patients who received any drug or prophylactic intervention to reduce risk of pancreatitis were excluded from the analysis 25 . Findings from the current study provide more robust data on pancreatitis rates, especially for elective procedures.

The present study is different than other analyses of volume effects on ERCP outcomes, in that only benign indications were included. A recent meta-analysis has suggested that higher-volume providers are more likely to be successful compared with lower-volume providers (OR 2; 95% CI 1.6–2.5) 10 ; however, a resulting impact on mortality was not observed. A population-based Swedish study reported reduced mortality at higher-volume centers, however, the threshold for a higher-volume center was only > 87 procedures per year, which is a lower threshold than might be expected in many developed countries 9 . In contrast, a recent large study suggested that lower mortality is related to a higher volume of cases of malignant biliary obstruction 7 . The current study found that an association with provider volume and all-cause mortality was highest in the highest-volume providers. However, the volume effect was not seen after excluding patients who required repeat ERCP within 90 days, which is a possible surrogate for more complex benign biliary disease. High-volume, particularly tertiary care, centers often receive referrals for more complex ERCP cases, including previously failed procedures, larger stones, and complex strictures. Such referral patterns may introduce referral bias and case-mix differences because these patients may have a higher baseline risk of complications and mortality. Therefore, the higher mortality observed in high-volume centers in our study should be interpreted cautiously and may not necessarily reflect differences in quality of care. It is also possible that despite excluding malignant and autoimmune biliary indications based on ICD-10 codes, there are residual confounders (e.g. indeterminate biliary strictures), which are not well coded in HES and may carry a higher risk of mortality.

The proportion of patients requiring emergency readmission, repeat ERCP within 90 days, or a repeat ERCP within the same admission is important information to explain to patients during procedure consent. Such information is often overlooked when compared with more immediate, severe AEs such as pancreatitis, bleeding, or death.

Although large observational studies are powerful tools to identify risks and associations within populations, there are a number of limitations to the present study. There is a potential for recording bias because accuracy of coded data depends on quality of medical records and on the staff coding the records. However, in the 2012/13 annual report on the quality of HES records, 99.3% of primary diagnoses and 99.9% of primary procedure codes were accurate 28 . Validation of ERCP codes in our study revealed that coding accuracy was above 96%. Therefore, we can be confident that the present study captures the vast majority of patients with benign indications for ERCP. Post ERCP pancreatitis and cholangitis rates were only reported for those who underwent ERCP as day cases and were subsequently admitted with these AE within 7 days because it was not possible to exclude reverse causality for patients who had ERCP as inpatients, because cholangitis and pancreatitis can be both indications for and AEs of ERCP. Therefore, it was not possible to assess rates of pancreatitis and cholangitis following inpatient ERCP for benign indications. This may introduce selection bias in estimation of these complication rates because outpatient ERCP procedures may represent a lower-risk population. Consequently, AE rates reported in this study may underestimate true overall complication rates. Future studies with access to clinical notes will be required to estimate rates of pancreatitis and cholangitis in patients who undergo ERCP as inpatients. Several potentially important procedure- and operator-related variables, including procedure complexity, endoscopist experience, use of rectal NSAIDs, and prophylactic pancreatic duct stent placement, were not available in the dataset. These factors may influence clinical outcomes and, therefore, residual confounding cannot be excluded. Future studies incorporating detailed procedure- and operator-level data are warranted to further clarify these relationships.

## Conclusions

In conclusion, this study demonstrates that short term all-cause mortality following ERCP for benign indications has fallen despite a substantial increase in the number patients with comorbidities and/or advanced age undergoing ERCP. All-cause mortality was associated with increasing age, males, increasing comorbidity, Black ethnicity, emergency ERCP, and increasing provider volume. Statistically significant variations in risk-adjusted 30-day all-cause mortality rates were found among ERCP providers.

## Supplementary Material

Supplementary Material Zusatzmaterial
