# Supplementary material for: All-cause mortality, adverse events and associated factors following endoscopic retrograde cholangiopancreatography for benign indications in England
Source: Endosc Int Open. 2026 Jun 8;14:a28653052. doi: 10.1055/a-2865-3052 (PMC13289825; doi:10.1055/a-2865-3052)

All-cause mortality, adverse events, and associated factors following endoscopic retrograde cholangiopancreatography for benign indications in England

Umair Kamran, Philip Harvey, Ben Coupland, Jemma Mytton, Kofi W Oppong, Nigel Trudgill

Supplementary Table 1

OPCS4 ERCP codes (all)

J38.1 Endoscopic sphincterotomy of sphincter of Oddi and removal of calculus HFQ

J38.2 Endoscopic sphincterotomy of sphincter of Oddi and insertion of tubal prosthesis into bile duct

J38.8 Other specified endoscopic incision of sphincter of Oddi

J38.9 Unspecified endoscopic incision of sphincter of Oddi

J39.1 Endoscopic sphincterotomy of accessory ampulla of Vater

J39.8 Other specified other therapeutic endoscopic operations on ampulla of Vater

J39.9 Unspecified other therapeutic endoscopic operations on ampulla of Vater

J40.1 Endoscopic retrograde insertion of tubal prosthesis into both hepatic ducts

J40.2 Endoscopic retrograde insertion of tubal prosthesis into bile duct NEC

J40.3 Endoscopic retrograde renewal of tubal prosthesis in bile duct NEC

J40.4 Endoscopic retrograde removal of tubal prosthesis from bile duct

J40.5 Endoscopic retrograde insertion of expanding covered metal stent into bile duct

J40.6 Endoscopic retrograde insertion of expanding metal stent into bile duct NEC

J40.7 Endoscopic retrograde renewal of expanding metal stent in bile duct

J40.8 Other specified endoscopic retrograde placement of prosthesis in bile duct

J40.9 Unspecified endoscopic retrograde placement of prosthesis in bile duct

J41.1 Endoscopic retrograde extraction of calculus from bile duct

J41.2 Endoscopic dilation of bile duct NEC

J41.3 Endoscopic retrograde lithotripsy of calculus of bile duct

J41.4 Endoscopic retrograde photodynamic laser therapy of lesion of bile duct

J41.8 Other specified other therapeutic endoscopic retrograde operations on bile duct

J41.9 Unspecified other therapeutic endoscopic retrograde operations on bile duct

J42.1 Endoscopic retrograde insertion of tubal prosthesis into pancreatic duct

J42.2 Endoscopic retrograde renewal of tubal prosthesis in pancreatic duct

J42.3 Endoscopic retrograde removal of calculus from pancreatic duct

J42.4 Endoscopic retrograde drainage of lesion of pancreas

J42.5 Endoscopic retrograde dilation of pancreatic duct

J42.8 Other specified therapeutic endoscopic retrograde operations on pancreatic duct

J42.9 Unspecified therapeutic endoscopic retrograde operations on pancreatic duct

J43.1 Endoscopic retrograde cholangiopancreatography and biopsy of lesion of ampulla of Vater

J43.2 Endoscopic retrograde cholangiopancreatography and biopsy of lesion of biliary or pancreatic system NEC

J43.3 Endoscopic retrograde cholangiopancreatography and collection of bile

J43.8 Other specified diagnostic endoscopic retrograde examination of bile duct and pancreatic duct

J43.9 Unspecified diagnostic endoscopic retrograde examination of bile duct and pancreatic duct

J44.1 Endoscopic retrograde cholangiography and biopsy of lesion of bile duct

J44.8 Other specified diagnostic endoscopic retrograde examination of bile duct

J44.9 Unspecified diagnostic endoscopic retrograde examination of bile duct

J45.1 Endoscopic retrograde pancreatography and biopsy of lesion of pancreas

J45.2 Endoscopic retrograde pancreatography and collection of pancreatic juice

J45.3 Endoscopic retrograde pancreatography through accessory ampulla of Vater

J45.8 Other specified diagnostic endoscopic retrograde examination of pancreatic duct

J45.9 Unspecified diagnostic endoscopic retrograde examination of pancreatic duct

ICD-10 codes for cancer

C17 Malignant neoplasm of small intestine

C22 Malignant neoplasm of liver and intrahepatic bile ducts

C23 Malignant neoplasm of gallbladder

C24 Malignant neoplasm of other and unspecified parts of the biliary tract

C25 Malignant neoplasm of pancreas

ICD10 codes for liver transplantation and autoimmune bile duct disease

Z94.4 Liver transplant status

K74.3 Primary biliary cirrhosis

K74.5 Biliary cirrhosis, unspecified

OPCS4 codes for complications of ERCP

Sedation complication codes

J69.0 Aspiration pneumonia NOS

J13 Pneumonia due to strep pneumonia

J14 Pneumonia due to Hemophilus influenzae

J15 Bacterial pneumonia, not elsewhere classified

J16 Pneumonia due to other infectious organisms, not elsewhere classified

J17 Pneumonia in diseases classified elsewhere

J18 Pneumonia, unspecified organism

J22 Unspecified acute lower respiratory infection

J96 Respiratory failure, not elsewhere classified

T88.6 - Anaphylactic reaction due to adverse effect of correct drug or medicament properly administered

T88.7 - Unspecified adverse effect of drug or medicament

Bleeding

K92.0 Hematemesis

K92.1 Melena

K92.2 Gastrointestinal hemorrhage, unspecified

I95 Hypotension

T81 Complications of procedures, not elsewhere classified

T81.0 Hemorrhage and haematoma complicating a procedure, not elsewhere classified

T81.1 Shock during or resulting from a procedure, not elsewhere classified

T81.7 Vascular complications following a procedure, not elsewhere classified

K91.84 Postprocedural hemorrhage and hematoma of a digestive system organ or structure following a procedure

K91.840 Postprocedural hemorrhage and hematoma of a digestive system organ or structure following a digestive system procedure

K91.841 Postprocedural hemorrhage and hematoma of a digestive system organ or structure following other procedure

K91.6 Intraoperative hemorrhage and hematoma of a digestive system organ or structure complicating a procedure

K91.61 Intraoperative hemorrhage and hematoma of a digestive system organ or structure complicating a digestive system procedure

K91.62 Intraoperative hemorrhage and hematoma of a digestive system organ or structure complicating other procedure

Perforation

T81.2 Accidental puncture and laceration during a procedure, not elsewhere classified

Y60.0 Unintentional cut, puncture, perforation or haemorrhage during surgical care

Y60.8 During other surgical and medical care

Y60.9 During unspecified surgical and medical care

K91.7 Accidental puncture and laceration of a digestive system organ or structure during a procedure

K91.71 Accidental puncture and laceration of a digestive system organ or structure during a digestive system procedure

K91.72 Accidental puncture and laceration of a digestive system organ or structure during other procedure

K22.3 Perforation, perforated (nontraumatic) esophagus

K63.1 Perforation of intestine (nontraumatic)

K66.1 Hemoperitoneum

K65.0 Generalized (acute) peritonitis

K65.1 Peritoneal abscess

K65.9 Peritonitis, unspecified

J85.3 Abscess of mediastinum

K83.2 Perforation of bile duct

Pancreatitis

K85.1 Biliary acute pancreatitis

K85.8 Other acute pancreatitis

K85.9 Acute pancreatitis, unspecified

K85.0 Idiopathic acute pancreatitis

K85.x Acute pancreatitis

Cholangitis

K83.0 Cholangitis

Other

T85 Complications of other internal prosthetic devices, implants and grafts

Supplementary Table 2 Thirty-day all-cause mortality rates after ERCP for benign indications performed between April 2017 and March 2020 stratified by sex and age.

	**Male**			**Female**			**Total**		
**Age group**	**Total patients**	**Mortality patients**	**Mortality rate**	**Total patients**	**Mortality patients**	**Mortality rate**	**Total patients**	**Mortality patients**	**Mortality rate**
18-49	3051	10	0.3%	8439	8	0.1%	11490	18	0.2%
50-59	3025	22	0.7%	4574	14	0.3%	7599	36	0.5%
60-69	4640	56	1.2%	5395	41	0.8%	10035	97	1.0%
70-79	6549	101	1.5%	7589	111	1.5%	14138	212	1.5%
80-89	4875	190	3.9%	7206	209	2.9%	12081	399	3.3%
≥ 90	836	49	5.9%	1753	89	5.1%	2589	138	5.3%
Total	22976	428	1.9%	34956	472	1.4%	57932	900	1.6%

ERCP, endoscopic retrograde cholangiopancreatography.

Supplementary Table 3 Multivariable logistic regression analysis of factors associated with 30-day mortality following ERCP for benign indications after excluding patients who underwent repeat ERCP within 90 days.

**Demographic category**		**Odds ratio**	**95% CI**	
Sex	Female	Reference category		
	Male	1.20	1.13	1.27
Age quintile	18-46	Reference category		
	47-62	3.46	2.70	4.43
	63-72	6.95	5.49	8.80
	73-81	11.51	9.15	14.48
	≥ 82	19.89	15.86	24.95
Deprivation	1 (most deprived)	Reference category		
	2	0.95	0.87	1.04
	3	0.94	0.86	1.02
	4	0.98	0.90	1.08
	5 (least deprived)	0.94	0.86	1.04
	Unknown	0.72	0.45	1.16
Ethnicity	White	Reference category		
	Black or Black British	1.43	1.05	1.96
	Asian or Asian British	1.10	0.94	1.30
	Mixed	1.29	0.70	2.37
	Other/unknown	2.65	2.35	2.98
Procedure type	Emergency	Reference category		
	Elective	0.29	0.27	0.32
	Other	1.44	1.16	1.78
Year of ERCP	2003/04	Reference category		
	2004/05	1.01	0.85	1.20
	2005/06	1.03	0.87	1.22
	2006/07	0.98	0.83	1.16
	2007/08	0.94	0.79	1.11
	2008/09	0.92	0.78	1.09
	2009/10	0.70	0.58	0.83
	2010/11	0.64	0.54	0.76
	2011/12	0.66	0.56	0.79
	2012/13	0.64	0.54	0.76
	2013/14	0.59	0.50	0.70
	2014/15	0.64	0.54	0.76
	2015/16	0.58	0.49	0.69
	2016/17	0.59	0.50	0.70
	2017/18	0.52	0.44	0.62
	2018/19	0.43	0.36	0.52
	2019/20	0.39	0.33	0.46
Charlson	0	Reference		

comorbidity score		category		
	1-4	1.25	1.14	1.38
	≥ 5	3.36	3.14	3.60
Provider annual ERCP volume	1-100	Reference category		
	101-150	0.94	0.87	1.02
	≥ 151	1.06	0.98	1.14

ERCP, endoscopic retrograde cholangiopancreatography.

Supplementary Figure 1 Yearly 30-day all-cause mortality rates post ERCP by age groups ERCP-Endoscopic retrograde cholangiopancreatography.

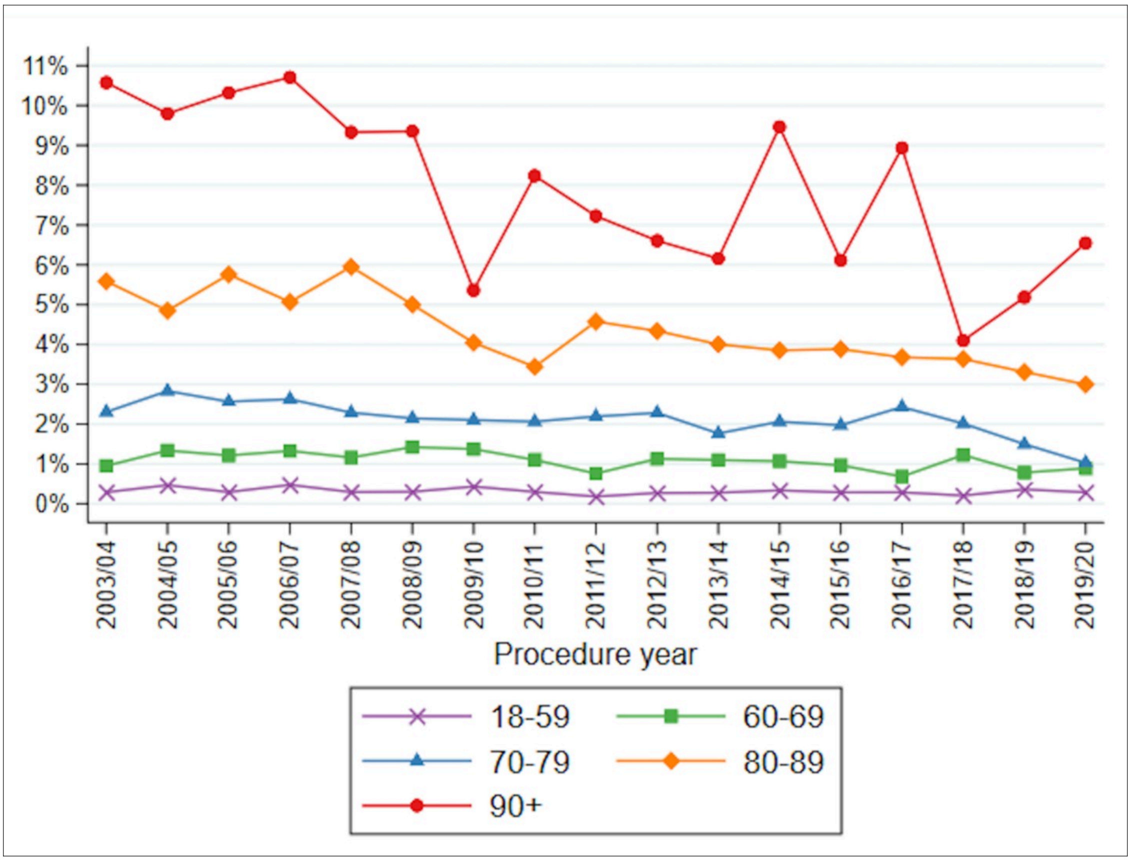

Supplement: Supplementary file 1 — Supplementary Material [file 10-1055-a-2865-3052_28705179.pdf]
